# *SULF 1* gene polymorphism, rs6990375 is in significant association with fetus failure in IVF technique

**Published:** 2015-04

**Authors:** Eskandar Taghizadeh, Seyed Mehdi Kalantar, Reza Mahdian, Mohammad Hasan Sheikhha, Ehsan Farashahi-Yazd, Saeed Ghasemi, Zahra Shahbazi

**Affiliations:** 1 *Department of Genetics, Shahid Sadoughi University of Medical Sciences, Yazd,Iran.*; 2 *Research and Clinical Center for Infertility, Shahid Sadoughi University of Medical Sciences, Yazd, Iran.*; 3 *Department of Molecular Medicine, Pasteur Institute of Iran, Tehran, Iran.*; 4 *Pasteur Institute of Iran, Tehran, Iran.*

**Keywords:** *SULF1 gene*, *Polymerase chain reaction*, *Restriction fragment length Polymorphysm*, *Single nucleotide polymorphism*, *Restriction endonucleases*, *rs6990375*

## Abstract

**Background:**

Sulfatase 1 (*SULF1*) function is to remove the 6-O-sulphate group from heparan sulfate. This action changes the binding sites of extracellular growth factors. *SULF1* expression has been reported to be changed in angiogenesis. We hypothesized that single nucleotide polymorphisms (SNPs) of *SULF1* would impact clinicopathologic characteristics.

**Objective:**

Study of *SULF1* gene polymorphism with fetus failure in in vitro fertilization (IVF) technique.

**Materials and Methods:**

We studied one common (minor allele frequency >0.05) regulatory SNP, rs6990375, with polymerase chain reaction and restriction fragment length polymorphism method, in 53 infertile women with fetus failure in IVF technique and 53 women with at least one healthy child as controls.

**Results:**

We found that rs6990375 is significantly associated with an early failure in IVF and frequency of G allele is high in women with fetus failure in IVF technique (p<0.001).

**Conclusion:**

These findings suggest that *SULF1*genetic variations may play a role in IVF technique fetus failure. Further studies with large sample sizes on *SULF1* SNPs may be useful in support of this claim.

## Introduction

In vitro fertilization (IVF) is often the best method of treatment for the couples that are suffering from infertility. In this method eggs are fertilized by sperms in vitro and then one of these fertilized eggs that it is in best quality, transfer in uterus (1-3). Implantation of embryo into the uterus is a critical stage in the establishment of pregnancy (4). Implantation is associates with secretion of a series of molecules including growth factors and morphogenesis. These factors cause blood to accumulate in uterus wall and angiogenesis to be occur (5). Some growth factors that are effective in this process are vascular endothelial growth factor, fibroblast growth factor, hepatic growth factor, Wingless-type MMTV integration site family member (WNT) and Sonic Hedghog (SHH) paths (6, 7).

Heparan sulfate proteoglycans (HSPs) are glycoproteins which have basic roles in regulating these signaling paths, and are able to affect growth at any stage (8). HSPs are present in the tissues of most multicellular organisms and act as helper receptor for growth factors and cytokines. Indeed these molecules act as internal regulators of cellular signaling paths and affect processes such as cellular growth proliferation/distinction and cellular immigration. Heparan sulfates (HS) have also basic roles in the primary stages of pregnancy (9,10). The sulfatase 1 (*SULF1*) enzyme is in the sulfatase group. *SULF1* gene is mapped on chromosome 8 (q 13.2-13.3) and its product is a protein with 871 amino acids (11).

The sulfatases function is to remove the 6-O-sulfate group from these proteoglycans. This action changes joining sites of signaling molecules and results in regulation and modification of the effects of heparan sulfate (12). The natural evolution of fetus depends on cellular interactions that are performed by a variety of molecules and different proteins. Several genetic studies on different spicies such as mice (rats) have proved the biological importance of proteoglycans such as heparan sulfates in the evolution, angiogenesis, morphogenesis and growth of fetus (13). One of the important specifications of Heparin sulfates is their desulfation pattern which is done by sulfatases including *SULF1* (14). This pattern has some effect on growth and cellular signaling such as Fibroblast Growth Factor (FGF), Vascular Endothelial Growth Factor (VEFG), Bone Morphogenic Protein (BMP), WNT, SHH, Gelial Cell Line-Derived Neurotrophic Factor (GDNF) and Heparin-Binding EGF-Like Growth Factor (HB-EGF) (15). Therefore, with respect to the role of growth factors and morphogenesis in the evolution stages of fetus and *SULF1* role in angiogenesis and implantation, in this research we have studied rs6990375 polymorphism of *SULF1* gene as a clinical polymorphism in infertile women with the background of at least once failure in IVF (16, 17).

We define fetus failure in IVF technique as a character in women that seem healthy but are infertile and are not pregnant despite using IVF and transferring at least two fetuses with good morphology. In other hand fetus failure in IVF technique in our study is infertility without known reason.

The goal of this project was *SULF1* gene polymorphism association study with fetus failure in IVF technique. The results may be useful for designing of some methods for IVF success improvement.

## Materials and methods

In this case control Study, all samples were collected from Research and Clinical Center for Infertility, Yazd, Iran from May to September 2013.

Cocran method was used for calculating sample volume. The cases were 53 infertile women with 20-37 years old who were not pregnant despite using IVF and transferring at least two fetuses with good morphology. The control group (n=53) were selected from 20- 37 year-old women with at least one healthy child and absence of endometriosis and uterus tubes blockage probability or other unknown infertility reasons.

Our study protocol was approved by the local ethics committee of Research and Clinical Center for Infertility, Yazd, Iran.

After giving the written informed consent, 5cc blood was obtained from all participants. Then DNA extraction was done manually with salting out method using cell lysis buffer, nuclei lysis buffer, proteinase K, ethanol, and some salts like Sodium Dodecyl Sulfate (SDS) and NaCl. DNA concentration, quality, and purity were measured using spectrophotometry.

**Figure 1:**
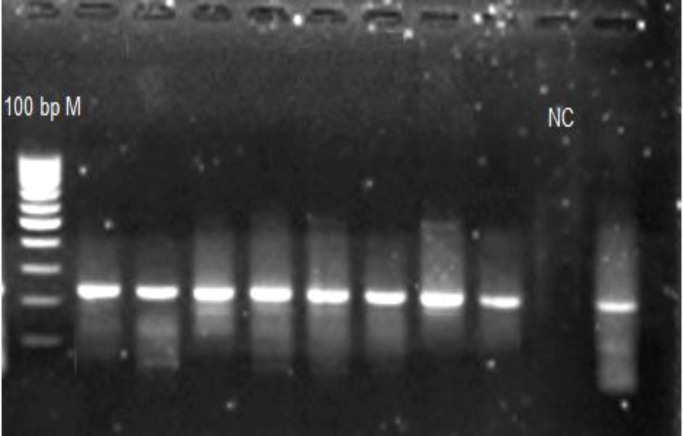
PCR products. Size of product is 277 bp. M: marker, NC: negative control

**Figure 2:**
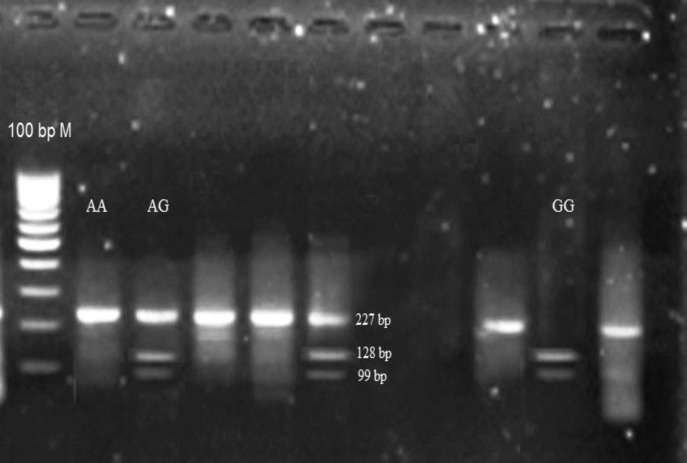
RFLP products in HhaI enzyme digestion. M: marker

Afterwards polymerase chain reaction (PCR) was done for SULF1 gene exon23 that contains rs 6990375 polymorphism and restriction length polymorphism (RFLP) was done with HhaI enzyme (Fermentas Company) according to table I, II. We controlled PCR and digestion product size with agaroze gel electrophoresis (Figure 1, 2). Demographic and clinical specifications of the cases were extracted from their clinical records and analyzed by statistical software. 

We analyzed case and control groups in some factors like age, type of infertility (primary or secondary), number of pregnancies, number of abortions, and number of failed transfers.

**Statistical analysis**

SPSS software (Statistical Package for Social Science, version 16.0, SPSS Inc, Chicago, Illinois, USA) and χ^2^ test was used for date analysis.

**Table I:** Polymerase chain reaction primer specifications

**Type**	**Sequence**	**GC%**	**Melting temperature (TM)**
Forward	5′ CCGCAGAACACCGAAGTAATT3′	47.6	55.5
Reverse	5′CCAGGGTAGCTTGGAATGTTG3′	52.4	55.9

**Table II:** Restriction fragment length polymorphism product size in different genotypes for Rs 6990375G>A

**PCR product size**	**A/A**	**G/A**	**G/G**
227	227	227 128 99	128 99

## Results

In total 106 women of two groups, 53 healthy women with at least one successful pregnancy and 53 women with the history of infertility were investigated. For reducing the effects of disturbing factors, the case and control groups were matched respecting the age and ethnicity. Table III shows demographic characteristics of the samples. The statistical variables that analyzed in this study are fertility and infertility, number of pregnancies, number of abortions and number of successful transfers (Table IV).

The results of screening the *Sulf1* rs6990375 G>A polymorphism gene variants among patients and healthy women, including the frequency of distribution of genotypes, are mentioned in table V. Our data showed that GG genotype was significantly higher among patients compared with control group (p<0.001). Of the 53 patients with infertility, the frequency of AA genotype was about same among test and control group respectively, 24.2% and 24.6% and GG genotypes was 33.3% for test group whereas this were 18.5% among controls. Therefore cases with GG genotypes were significantly more susceptible to infertility. While heterozygous genotype, AG was 56.9% in healthy women, compared with 42.4% in patients, which is significantly related to the chance of successful pregnancy.

**Table III:** Demographic characteristics of the case and control groups

**Demographic variables **	**Case group**	**Control group**
	**Frequency n (%)**	**Frequency n (%)**
Type of infertility		
Primary	30 (56.6)	-
Secondary	23 (43.4)	-
Age (years)		
	14 (26.4)	15 (29.2)
26-30	16 (30.2)	23 (43.1)
>30	23 (43.4)	14 (26.7)
Number of pregnancy		
1	16 (30.2)	15 (27.7)
2	3 (5.7)	15 (29.2)
3	1 (1.9)	11 (21.5)
>4	1 (1.9)	11 (21.5)
No pregnancy	32 (60.4)	0 (0)
Number of abortion		
0	34 (64.2)	45 (84.6)
1	13 (24.5)	6 (10.8)
2	5 (9.4)	1 (1.5)
6	1 (1.9)	1 (1.5)
Number of unsuccessful transfers		
2	27 (50.9)	-
3	17 (32.1)	-
4	5 (9.4)	-
5	4 (7.5)	-

**TableIV:** χ2 coefficient for GG genotype correlation with other variables

**Independent Variables **	**χ** ^2 ^ **coefficient**	**Degree of freedom (Df )**	**p-value**
Type of infertility	6.233	2	0.019
Number of pregnancy	12.011	8	0.141
Number of failed transfers	8.609	8	0.344
Number of abortions	29.211	6	0.001

**Table V:** Single nucleotide polymorphism (SNP) genotype frequency

**Genotype**	**Case group** **Frequency n(%)**	**Control group** **Frequency n(%)**
AG	22 (42.4)	30 (56.9)
AA	13 (24.2)	13 (24.6)
GG	18 (33.3)	10 (18.5)

## Discussion

The aim of present case-control research was to study association of the *SULF1* gene polymorphism (rs6990375G>A) with increased risk of fetus failure in IVF technique among patients referred to Research and Clinical Center of Infertility, Yazd, Iran. Samples of this study were collected from patients that didn’t have known problems in clinical examination, and seem healthy but they were infertile women who were not pregnant despite using IVF and transferring at least two fetuses with good morphology. In other hand, their infertility reason wasn’t known. Other infertile women that have known infertility problems were excluded from our study.

Altogether our exclusion and inclusion criteria was infertility despite using IVF with transfer of at least two good embryo inmorphology, maximum age of 37 years old and absence of endometriosis and uterus tubes blockage probability or other unknown infertility reasons for sample group and having at least one natural pregnancy for control group. Previous studies have shown that suffering from fetus failure in IVF technique is a multifactorial condition. Some factors in this condition have been examined, and genetic factors are one of them (18). The *SULF1* product is a transmembrane protein which acts as a post transcriptional modifier (19). This protein action is to delete or modify 6-O-sulfate group from proteoglycans such as heparin sulphate. This modification has an important role in alteration of binding site of ligands and molecules such as FGF, VEGF, BMP, WNT, SHH, etc., and by this action it affect several cellular processes such as angiogenesis, cellular proliferation, wound regeneration and healing and fetal evolution (12).

Then *SULF1* are arylendosulfatase which alter the sulfation patterns of proteoglycans and the binding site of many growth factors (18). They have an important role in embryogenesis. An important nutrient for human growth and development is Sulfate (SO_42−_) which is obtained from the diet. In addition, the intra-cellular metabolism of sulfur-containing amino acids such as methionine and cysteine can produce sulfate. But during pregnancy, fetal tissues cannot produce enough sulfate, therefore the fetus must obtain the sulfate from the maternal circulation. In recent years, there is a growing interest in sulfate and its role in fetal development (20).

Previous studies have investigated the effects of reduction of *SULF1* and Sulfatase 2 (*SULF2)* in abnormal embryonic development (15-17). We think that *SULF1* polymorphisms may have an effect on occurrence of fetus failure in IVF technique. In order to assess the above hypothesis, rs6990375 SNP frequencies were calculated using PCR-RFLP among two groups included women that had fetus failure in IVF technique as case group and healthy women as control group. This is the first report of *SULF1* gene polymorphisms association with Fetus failure in IVF technique. The most studies that have been conducted so far on this gene, has focused on its role in cancer such as ovary, breast, stomach and hepatocellular cancer. In these studies *SULF1* gene expression was altered in cancerous tissues due to its role in regulating angiogenesis. The study that conducted by Holst *et al* shows the role of *SULF1* gene in fetal evolution. In their study rat *SULF1* gene knock outing caused reduction in fetus survival (13). Also a study conducted by Lum *et al* showed that *SULF1* and *SULF2* genes product lack or reduction causes evolutionary, neural, skeletal and muscular disorders (21).

The survey about polymorphisms of *SULF1* gene, for the first time was performed by Han *et al* who genotyped five common single nucleotide polymorphisms (SNPs) in *SULF1* gene to evaluate associations between these functional *SULF1* SNPs and the risk of ovarian cancer (18). They found that some of the *SULF1* gene SNPs can have one of the following predicted functionalities: 1) affecting transcription factor binding sites (TFBS) activity in the putative promoter region, 2) affecting splicing activity, or 3) affecting the microRNA binding sites activity. According to their study the rs6990375 is a functional SNP affecting the micro RNA binding site activity (18). Recently numerous studies have shown that miRNAs have important role in the occurrences of gynecological disorders such as endometriosis, preeclampsia, infertility and recurrent miscarriages. The targets of many miRNAs are molecules that are closely associated with processes such as cellular proliferation and differentiation.

Because *SULF1* as a regulator is associated with many signaling molecules such as BMPs, fibroblast growth factors (FGFs), hedgehogs, and Wnts, therefore it is a possibility that *SULF1* can be one of these target molecules (22). Heparan sulfate proteoglycans (HSPGs), when sulfated, serve as co-receptors for many growth factors and cytokines. *SULF1* desulfate cellular HSPGs. Therefore *SULF1* seems to modulate growth factor and cytokine signaling. In facts, *SULF1* inhibits the co-receptor function of HSPGs in different growth factors such as fibroblast growth factor 2, vascular endothelial growth factor, hepatocyte growth factor, PDGF, and heparin-binding epidermal growth factor (HB-EGF).

It was shown that forced expression of *SULF1* decreases cell proliferation, migration, and invasion (23). In summary, our findings reveal a new genetic association between occurrences of fetus failure in IVF technique and *SULF1* gene mutation. But our study had some limitation therefore we suggest doing further genetic studies with a larger sample size to confirm and clarify this association. Investigating *SULF1* gene expression patterns in patients with fetus failure in IVF technique, and also study of overlapping *SULF1* and *SULF2* roles in development could help this confirmation. Our research is the first study that investigates *SULF1* role in infertility. Results show significant differences between case and control groups in genotype and alleles frequencies of this SNP. First, increase in the frequency of G allele in the test group, second, increase in the GG genotype frequency in the test group, third, a significant correlation between GG genotype and infertility type (primary or secondary), and fourth, a significant correlation between GG genotype and number of abortions. These results may show *SULF1* enzyme role in the fetus evolutionary stages and limbs growth. This finding supports Lum *et al* study results.

The rs6990375 polymorphism occurs in the region of *SULF1* gene that code for micro RNA binding domain. Results show that GG genotype make sulphatase enzyme more vulnerable to micro RNA destruction (19, 24). Women with this genotype are exposed to increased risk of evolutionary and nesting disorders. Women with GA heterozygote genotype have more chance for successful pregnancy than homozygote genotypes.

## Note

***This article extracted from M.Sc. thesis. (Eskandar Taghizadeh)***
